# Participation and Refusal in an Implementation Trial: Insights from the Accelerating Cervical Cancer Elimination through the Integration of Screen-and-Treat Services (ACCESS) Study

**DOI:** 10.21203/rs.3.rs-9798338/v1

**Published:** 2026-06-11

**Authors:** Stanley Chinedu Eneh, Ngozi Idemili-Aronu, John Oluwaseyi Jemisenia, Babayemi Oluwaseun Olakunde, John Olajide Olawepo, Ijeoma Uchenna Itanyi, Gregory Aarons, Echezona Edozie Ezeanolue

**Affiliations:** IVAN Research Institute, University of Nigeria Nsukka; IVAN Research Institute, University of Nigeria Nsukka; IVAN Research Institute, University of Nigeria Nsukka; IVAN Research Institute, University of Nigeria Nsukka; IVAN Research Institute, University of Nigeria Nsukka; IVAN Research Institute, University of Nigeria Nsukka; Department of Psychiatry, University of California San Diego; IVAN Research Institute, University of Nigeria Nsukka

**Keywords:** Clinical trial recruitment, HIV, Cervical Cancer, patient decision-making, implementation trial, Nigeria

## Abstract

**Background:**

Patient recruitment and retention are major challenges in efforts to diversify clinical trials, particularly in resource-limited settings. Understanding factors that influence participation is critical for effective trial implementation. This study explores factors influencing participation and refusal in the Accelerating Cervical Cancer Elimination through the integration of Screen-and-treat Services (ACCESS) implementation trial among women living with HIV (WLHIV) in Nigeria.

**Methods:**

A phenomenological qualitative approach was employed. In-depth interviews were conducted with 21 participants (12 acceptors, 9 decliners), purposively sampled from 12 NISA-MIRC HIV treatment facilities across Nigeria’s six geopolitical zones. Interviews were conducted in English, Hausa, or Yoruba and translated for thematic analysis. Data was analyzed using comparative thematic analysis to identify cross-cutting domains influencing decision-making.

**Results:**

Nine themes emerged, including comprehension of information, fear and risk perception, perceived benefits and opportunities, logistical barriers, willingness to participate in the future, suggestions for improvement, privacy concerns, and preferred recruitment approaches. Acceptors overcame concerns through clear communication, emotional reassurance, trust, and perceived personal relevance. Decliners experienced “barrier dominance,” where unresolved fears, logistical constraints, mistrust, and poorly timed recruitment outweighed potential benefits. Both groups suggested improvements, such as enhanced communication, community outreach, confidentiality assurances, and logistical support.

**Conclusions:**

Trial participation decisions reflect distinct cognitive, emotional, and structural pathways. Patient-centered communication, culturally appropriate explanations, trust-building, and logistical support are critical to improving trial recruitment and retention. Addressing these factors can enhance equitable participation and strengthen the quality and generalizability of trial evidence in Nigeria and similar low-resource contexts.

## Background

Globally, patient recruitment for clinical trials faces substantial barriers [1]. Commonly reported barriers include a limited pool of eligible participants, high procedural and time demands on patients and investigators, inadequate institutional and logistical support for trial staff, and restrictive eligibility criteria that exclude otherwise suitable participants [2, 3]. Studies have highlighted the impact of patient recruitment failure resulting to trials delays, statistical power reduction, trial timeline extension significant cost implications and sometime leading to compromise trials [3–6]. Randomized controlled trials (RCTs) are considered the gold standard for generating robust scientific evidence on the efficacy of drugs and interventions, and clinical practice guidelines predominantly rely on evidence derived from these trials [7, 8]. Consequently, patient recruitment and retention are essential to the successful implementation trials. Therefore, enhancing trial participation would significantly broaden the evidence base, increase the representatives of scientific findings, and improve the generalizability of results, thereby benefiting a wider spectrum of patients [9]. Investigative efforts are still hindered by inadequate accrual, and slow recruitment rates which might cause studies to last longer or even end crucial clinical studies early.

Although barriers to recruitment in randomized controlled trials have been extensively studied, the literature has tended to focus on discrete factors, such as age, socio-economic status, and logistical constraints, rather than exploring how these barriers collectively shape decisions to participate or refuse [3, 7, 10–21].

However, the RCTs landscape in Nigeria is complex and influenced by a variety of socio-economic, cultural, and infrastructural factors as well as patient accrual [22, 23]. Studies highlighted the impact of patient recruitment failure resulting to trials delays, statistical power reduction, trial timeline extension significant cost implications and sometime leading to compromise trials [3–6]. However, recruitment into RCTs continues to be challenging. Historical incidents, like the Pfizer Trovan trial controversy in Kano-Nigeria (1996), have fueled ongoing skepticism about RCTs in Nigeria [24, 25].

Despite the well-documented barriers to patient accrual in randomized controlled trials (RCTs) in Nigeria, most existing studies have focused on clinical (therapeutic) trials, with limited data on implementation trials [25, 26]. Unlike clinical trials, which evaluate the effects of treatments or interventions on patient health outcomes, implementation trials assess the effectiveness of strategies designed to support the adoption, integration, and sustainability of evidence-based interventions in real-world settings [26].

Therefore, in the context of the Accelerating Cervical Cancer Elimination through the integration of Screen-and-treat Services (ACCESS Study), an implementation trial integrating cervical cancer screening into HIV care [27], participant refusal during recruitment highlighted a critical gap in the literature: the limited understanding of why eligible women living with HIV decline participation in real-world implementation trials.

The ACCESS Study aims to identify effective implementation strategies to improve the integration of cervical cancer screening and treatment services into existing HIV programs in Nigeria [27]. With a population over 200 million, Nigeria presents a significant potential site for RCTs. The nation’s diversity and the widespread occurrence of various infectious and non-communicable diseases make it a crucial location for medical research [28]. While data on clinical trial recruitment and retention in resource limited settings is growing, evidence on implementation trial accrual remains limited [29–31]. Thus, this study aimed to understand factors associated with participation and refusal to participate in the ACCESS Study, an implementation trial evaluating strategies to improve the integration of cervical cancer screening and treatment services into existing HIV programs in Nigeria [27].

## Methodology/Materials

### Study Design

A phenomenological approach was used to gain insights into factors associated with participation and refusal in implementation trial in Nigeria. A phenomenological approach was deemed suitable for our study as a qualitative research methodology focused on exploring and understanding individuals experience and making sense of a particular phenomenon or event [32]. In-depth interviews (IDIs) were conducted with patients who were enrolled in the ACCESS Study and those who were approached but did not enroll [27]. Participants provided their phone numbers to allow follow-up at their next clinic visit.

### Study Setting and Participants

The ACCESS Study was a cluster randomized hybrid type III implementation trial in Nigeria that leveraged 12 comprehensive HIV treatment facilities across the country’s six geopolitical zones, with the full study design described elsewhere [27]. These facilities were designated as Nigeria Implementation Science Alliance (NISA) Model Innovation and Research Centers (NISA-MIRC) [33] that are part of the ICON-3 Practice-based Research Network, which includes 12 community centers, 21 NISA-MIRCs and 6 Regional Centers of Excellence. Collectively, these sites manage over 60,000 WLHIV receive care at these sites [33]. This study focused on women living with HIV (WLHIV) who were successfully enrolled in the ACCESS Study as well as those who declined to participate when approached during the enrollment phase. These participants indicated that they might reconsider participating in the trial at a subsequent clinic visit and provided their phone numbers for follow-up.

### Participants Selection

Patient enrollment for the ACCESS Study took place between November 13, 2023, and May 31, 2024, at 12 NISA-MIRC comprehensive HIV treatment facilities across Nigeria's six geopolitical zones. During the enrollment process, WLHIV were enrolled in the study, while some declined participation in the initial trial phase. Refusals/decline occurred across five of the six geopolitical zones. In total, over 4,000 WLHIV were enrolled in the ACCESS Study [27], and 63 patients were approached but did not enroll.

### Sampling Techniques

Participants were selected using purposive sampling from geopolitical zones where non-participation in the study had been reported. To capture a wide range of experiences and opinions on the issue under investigation, maximum variation purposive sampling was utilized, taking into account the number of potential participants in the study. This strategy aimed to capture a diverse array of perspectives among relevant participants. In qualitative research, purposive sampling is commonly used to intentionally recruit individuals with characteristics, experiences, or perspectives relevant to the research question.

In this study, WLHIV who initially declined participation were recruited from five geopolitical zones where refusals had occurred. In addition, two participants were recruited from each of the six geopolitical zones represented in the ACCESS Study among individuals who had agreed to participate. A total of 21 WLHIV were enrolled in this study, including participants who initially declined participation (n = 9) and participants enrolled in the ACCESS Study (n = 12). This approach ensured the collection of the most pertinent and insightful information from both categories [34].

### Data collection

Qualitative data were collected by 12 Site Research Coordinators (SRCs) trained in qualitative data collection and received additional training on the study objectives to enhance their content knowledge. This protocol training was conducted by the ACCESS Study Team and investigators, focusing on data collection through in-depth interviews (IDIs) as the primary method over the telephone. The IDI questions were translated into English, Hausa, and Yoruba to account for language differences across Nigeria and to facilitate communication with study participants. The SRCs translated all collected data into English for analysis. Qualitative data were collected through telephone interviews, with participants’ consent to record the conversations. Telephone interview was chosen because it offered flexibility regarding the time and location of the interviews and increased anonymity, consistent with prior studies [34–38].

### Data Management and Analysis

Data were analyzed using a Comparative Thematic Analysis approach, integrating principles from the Framework Method and constant-comparative techniques to systematically compare patterns between women who accepted and those who declined cervical cancer screening. Following Braun and Clarke’s [39, 40] six phases of thematic analysis, familiarization, coding, theme development, review, definition, and reporting, transcripts were read repeatedly and inductively coded to capture semantic and latent meanings. Codes were then organized into analytic matrices that contrasted ‘acceptors’ (those who accepted to participate in the study) and ‘decliners’ (those who declined to participate in the study) across shared themes, allowing systematic examination of similarities, divergences, and explanatory mechanisms, consistent with comparative analytic traditions described by Gale et al. [41] and Glaser & Strauss [42]. Through iterative comparison, higher-order themes were refined to reveal two distinct but intersecting decision-making pathways. Reflexive practice was maintained throughout to ensure analytic rigor, coherence, and interpretive transparency [43].

## Results

### Demographic Characteristics of Participants

As shown in Table 1, participants were mostly aged 40–49 years, with acceptors more represented in the younger age group (30–39 years) and decliners more in the 40–49 group. The majority in both groups were married in monogamous relationships. Most acceptors had post-secondary education, while decliners were more distributed across lower education levels. Most participants were Christian, although a slightly higher proportion of decliners identified as Muslim.

### Thematic Findings

The analysis generated nine cross-cutting themes, each comprising divergent sub-themes for two categories we labelled as: *Acceptors* and *Decliners*. Comparison of these two participant groups offers critical insight into how women interpreted the invitation, evaluated personal risk, navigated emotional and logistical barriers, and made participation decisions (summarized in Table 2).

The analytic approach revealed that acceptors and decliners did not merely differ in degree (e.g., “more informed” or “less informed”); rather, they operated within distinct cognitive-emotional frames, shaped by the quality of information received, interpersonal experience, and broader structural realities. While acceptors often demonstrated “barrier transcendence”, which is the ability to move past concerns through support, clarity, or motivation, decliners illustrated “barrier dominance,” where constraints compounded and overshadowed potential benefits. The following themes are presented comparatively to illustrate these divergent participation pathways.

### Information, Awareness and Comprehension

For acceptors, communication was experienced as clear, calm, and enabling, building initial confidence and fostering comprehension. Explanations were described as detailed yet accessible, often delivered in supportive interpersonal tones. This process developed a sense of partnership rather than instruction, with women repeatedly expressing how information empowered them to make informed choices. As one participant noted how the approach itself facilitated understanding: *“It’s only your approach and the way you explain… the way you calmly explained about the study made me to also join.”* (**Acceptor, Annunciation Specialist Hospital, Enugu**)

Information was not merely received; it was absorbed, transformed into personal knowledge, and even relayed to others. Acceptors often expressed a sense of having “gained,” “learned,” or “added knowledge,” demonstrating cognitive moves that reinforced participation.

On the contrary, those who declined commonly reported that information was too fast, incomplete, or not contextualized. Communication was experienced as rushed or poorly timed, leaving women confused or doubtful. For many, the use of mixed languages without ensuring comprehension created additional disconnect. As one woman explained: *“Hmm… not really. You explained, but it was fast. You were mixing English and Hausa, and I didn’t want to look like I don’t understand.”* (**Decliner, General Hospital Funtua, Kastina**). Another admitted that she simply did not grasp the purpose: *“I feel like it’s not clear… I don’t understand that much.”* (**Decliner, Oron General Hospital, Akwa Ibom**).

This “information disconnection” created a cognitive barrier where uncertainty or incomplete understanding made refusal the safer option. Unlike acceptors, who interpreted information as empowering, decliners interpreted it as inadequate, risky, or irrelevant.

The divergent information experiences between the acceptors and decliners suggest that quality of communication was a primary determinant of participation. Acceptors benefited from slow, calm, relational communication, while decliners encountered fast, transactional, or poorly timed explanations. The same content, when delivered differently, produced contrasting levels of comprehension and trust.

### Fear, Risk Perception, and Emotional Responses

Acceptors were not free of fear at the beginning; many initially felt anxious about cancer, the screening process, or the possible results. However, structured education and counselling played a transformative role. Fear became manageable, even constructive, reinforcing the need to “know one’s status.” A participant who initially feared because of her positive result described how counselling shifted the experience: *“It was not easy… when I discovered, I am positive, it was not easy. But counselling helped me overcome the self-stigma.”* (**Acceptor, Dalhatu Araf Specialist Hospital, Lafia, Nasarawa**).

Others emphasised that reassurance mitigated dread: *“It erased fear and gave confidence to go for screening at any time.”* (**Acceptor, General Hospital Funtua, Kastina**). This progression from apprehension to reassurance highlights the essential role of emotional support in shaping participation.

On the other hand, decliners operated within an unresolved fear frame, shaped by fear of cancer, fear of receiving bad news, fear of bodily exposure, fear of being singled out, and fear that was intensified by incomplete information. Without adequate emotional support, fear became an avoidance mechanism rather than a motivator. For example, one participant voiced this vividly: *“When I hear ‘cancer screening,’ my heart beats fast… I didn’t want bad news.”* (**Decliner, General Hospital Funtua, Kaduna**).

Another participant expressed the psychological overwhelm of mixing HIV-related distress with additional testing: *“This my condition [HIV] is not something I want to talk to people all the time.”* (**Decliner, Oron General Hospital, Akwa Ibom**).

While acceptors experienced fear as an initial hurdle that could be worked through, decliners experienced fear as a barrier that halted engagement. Supportive communication enabled acceptors to reinterpret their fear as motivation to act. Decliners, who received incomplete or hurried explanations, did not receive the emotional support needed to move past their worries. As a result, fear produced two distinct behavioral pathways: fear that was managed and led to participation, and fear that remained unresolved and resulted in avoidance.

### Health Motivation, Personal Relevance, and Prevention Beliefs

Acceptors often understood screening as part of a broader commitment to caring for themselves, particularly given their HIV status. Many recognized their heightened vulnerability to other illnesses and saw screening as essential for maintaining their health and protecting their families. As one participant remarked: *“I accepted it because I wanted to know more about my health.”* (**Acceptor, General Hospital Alimosho, Lagos**). Additionally, for some, the status of the disease itself heightened perceived risk, leading them to accept participation: *“Because of the status… most of the women are vulnerable to anything.”* (**Acceptor, Calabar General Hospital, Cross River**). This orientation reflects a preventive mindset where health maintenance is seen as an active responsibility.

However, decliners often rationalized refusal through low perceived susceptibility: *“I’m above the age of 49… I’m not sexually active… so there is no need.”* (**Decliner, Calabar General Hospital, Cross River**).

### This perception reveals misconceptions about age and cervical cancer risk.

Some decliners assumed screening was irrelevant to them personally, thereby deprioritizing participation. Others equally considered it an unnecessary burden or intrusion, specifically when heightened by fatigue, stress, or social obligations.

Acceptors viewed screening as personally meaningful, integrating it into their sense of responsibility for their own wellbeing. In contrast, decliners tended to see screening as something that is not for them, distancing themselves from perceived eligibility or relevance. This difference was not driven by factual knowledge alone but by how health information was absorbed, interpreted, and connected to one’s identity, risk perception, and everyday realities. For acceptors, screening represented an opportunity to safeguard their health and gain clarity. For decliners, however, it appeared as an unnecessary or burdensome task. This divergence highlights how meaning-making, rather than mere awareness, shaped the direction of their decisions.

### Perceived Benefits and Opportunities

Acceptors consistently identified a mix of educational, psychological, social, and practical benefits. These included reassurance, new knowledge, empowerment to educate others, contributing to research, and receiving free services. As an example, one participant summarized this positive framing: *“My experience is very educational. I’m learning so many new things.”* (**Acceptor, Calabar General Hospital, Cross River**). Another accepting participant noted: *“I participated because I know fully about this cancer screening and at the end of the day I will tell my colleagues about it… as in my peer group.”***(Acceptor, General Hospital Billiri, Gombe)**. Overall, acceptor**s** frequently perceived their participation as contributing to community uplift, future research, and wider public health.

Decliners, by contrast, reported muted or absent perception of benefits. Even when acknowledging the potential usefulness of screening in theory, they did not internalize its benefit for themselves. As one participant remarked: *“I know screening is good… but I didn’t think about the benefit that day because I was already stressed.”* (**Decliner, General Hospital Funtua, Kaduna State**). Others expressed skepticism about program continuity: “*I feel like programs are coming, this one brings this, this one brings that. Before we know, off they go, and nothing is being done about it*.” **(Decliner, Dalhatu Araf Specialist Hospital, Nasarawa)**.

Where acceptors recognized both tangible and intangible benefits, decliners perceived little to gain and much to lose in terms of time, privacy, and emotional ease. These contrasting assessments of value were pivotal as women with a high sense of benefit were more likely to participate, while low benefit perception contributed directly to refusal.

### Practical, Logistical, and Time-Related Barriers

Despite encountering real logistical challenges, transport costs, long waiting times, and occasional shortages of screening supplies, acceptors largely framed these constraints as manageable inconveniences rather than reasons to withdraw. Several women highlighted transportation difficulties or the financial pressures associated with clinic attendance, noting that *“some don’t have transportation”* (**Acceptor, Calabar General Hospital, Cross River**) and that *“financial support… for drugs, food, and transport… would help younger girls”* (**Acceptor, Calabar General Hospital, Cross River**). Others pointed to systemic issues in service delivery, such as instances when screening could not proceed because *“there are no screening consumables to be screened”* **(Acceptor, General Hospital, Billiri, Gombe**). Yet, even when these limitations disrupted the process, they were described with frustration, not discouragement. For most acceptors, the importance of cervical screening outweighed these logistical burdens, reflecting a pattern of barrier transcendence grounded in motivation and perceived personal value. As one participant observed, *“Time management will equally be of benefit”* (**Acceptor, Mother of Christ Specialist Hospital, Ogui, Enugu**), signaling a desire for improvement rather than an intention to disengage.

For decliners, logistical barriers were overwhelming and became the primary reason for refusal. Many described severe time pressure, arriving at the clinic already exhausted from long waits and needing to rush back to work or pick their children from school. As one explained, *“I was in a hurry… running back to work… I didn’t have time to listen very well. I was in a hurry”* (**Decliner, Faith Alive Foundation, Plateau**). Others emphasised distance and financial strain, noting that *“the place I come from is far… I borrow money to come here”* (**Decliner, Mother of Christ Specialist Hospital, Ogui, Enugu**). Transport costs, competing responsibilities, and clinic delays combined to make participation feel unrealistic, turning these practical difficulties into decisive barriers that overshadowed any perceived benefit of screening.

The contrast between both groups was clear. Acceptors regarded logistical barriers as manageable inconveniences that could be worked around, whereas decliners experienced the same challenges as insurmountable obstacles that made participation unrealistic. For decliners, time scarcity, fatigue, transport costs, and financial strain were not minor backdrop issues but powerful explanations that actively shaped refusal. These pressures compounded their daily burdens, making screening feel impractical despite any interest or awareness they might have had.

### Privacy, Confidentiality, and Bodily Exposure Concerns

Acceptors acknowledged some initial concern about exposure or data handling, but these worries were quickly eased by the reassurances provided by staff. Statements such as *“Yes, I will be open to participate as long as you assure confidentiality”* (**Acceptor, Annunciation Specialist Hospital, Emene, Enugu**) and *“I am confident… the clinic manager is aware”* (**Acceptor, Dalhatu Araf Specialist Hospital, Nasarawa**) reflect a level of trust in healthcare workers that neutralized anxiety about genital examination or information sharing. This trust created a sense of safety, allowing acceptors to view confidentiality as adequately protected.

Decliners, however, expressed deeper and more persistent concerns. Many worried about who might access their information, asking, *“Who will see my details?”* (**Decliner, General Hospital Funtua, Kastina**). Others voiced broader mistrust in health staff, fearing that personal information would be mishandled or discussed informally: *“You people like to use somebody to do study… after you carry it and discuss it with your family members”* (**Decliner, Mother of Christ Specialist Hospital, Ogui, Enugu**). Some emphasised a preference for strict personal privacy, stating, *“It’s not every information you give out I feel is confidential.”* (**Decliner, Dalhatu Araf Specialist Hospital, Nasarawa**). Overall, privacy concerns were shaped not by exposure alone but by varying levels of trust. For acceptors, trust reduced anxiety; for decliners, mistrust amplified it and contributed to refusal.

### Recruitment Approach and Social Interaction

Interpersonal approach strongly shaped women’s willingness to participate, with acceptors and decliners describing markedly different experiences. Acceptors consistently highlighted the importance of being approached with respect, calmness, and clarity. Many described feeling valued, listened to, and treated with dignity. As one woman explained, *“I didn’t have much thought, it’s only your approach and the way you explained to them while I was waiting to be attended to; so I decided to join. The way you calmly explained about the study made me to also join* (**Acceptor, Annunciation Specialist Hospital, Emene, Enugu**). Among several acceptors, the style of communication, gentle, patient, and supportive, was repeatedly described as central to their decision. Peer engagement also played a role, with some noting that *“it was the way other people were participating that made me join”* (**Acceptor, Annunciation Specialist Hospital, Emene, Enugu**), indicating a likely social reinforcement effect where group dynamics encouraged acceptance.

In contrast, decliners often recalled being approached at moments of exhaustion and or urgency. Several described long clinic waits followed by a hurried interaction, such as *“I was in a hurry and tired… I have really stayed long in the facility and it was time for me to pick my children from school”* (**Decliner, Annunciation Specialist Hospital, Emene, Enugu**) or *“I didn’t have time to listen”* (**Decliner, Dalhatu Araf Specialist Hospital, Nasarawa**). Being addressed when fatigued or rushing to meet other obligations made the invitation feel intrusive or poorly timed, reducing their willingness to engage regardless of tone. Summarily, the findings demonstrate that both the manner and timing of recruitment matter. They implied that a respectful approach could encourage participation, but even a friendly tone cannot offset poor timing or participant fatigue.

### Suggestions for Improvement

Acceptors generally proposed progressive improvement aimed at broadening reach and increasing participation. They emphasised the value of community-based outreach, involvement of husbands, better communication strategies, and small incentives. For example, several participants recommended expanding screening beyond facilities to villages and PHCCs, noting that *“the means of communicating to people… should reach those in the villages”* **(Acceptor, General Hospital, Billiri, Gombe**). Others suggested providing free medications, transport assistance, or modest financial support, explaining that such incentives would make participation easier and more attractive. Some highlighted the importance of involving spouses to strengthen decision-making support.

Decliners, in contrast, emphasised remedial changes to address the practical and communication barriers that had prevented their participation. Many requested early notifications: *“inform that person ahead of time… two weeks, three weeks, a month”* (**Decliner, Dalhatu Araf Specialist Hospital, Nasarawa**), as well as language-appropriate explanations and reduced waiting times. Transport reimbursement and confidentiality assurances were also key: *“Call people before their clinic day. Explain in Hausa properly. Don’t rush”* (**Decliner, General Hospital, Funtua, Kastina**). In all, acceptors focused on scaling and added value, while decliners prioritised eliminating barriers that had previously made participation unfeasible.

### Future Participation Willingness

Both groups expressed some openness to future participation, but the nature and strength of this willingness differed considerably. Acceptors generally conveyed strong enthusiasm, often linking their willingness to the value they derived from the current study. Several noted they would readily participate again if supportive measures such as free medication or treatment were provided, with one stating, *“Yes, … I'm willing… providing that free medication, free treatment will actually go a long way”* (**MOCSHE, Acceptor**). Others framed future participation as an opportunity to help peers, reflecting a sense of shared benefit and community responsibility.

Decliners, however, demonstrated more cautious and conditional openness. Their willingness depended on resolving practical barriers, particularly scheduling and transportation challenges. As one explained, *“Yes, if there was a way to schedule it on another day… or transport support”* (**Decliner – Follow-Up**). Overall, acceptors showed motivated re-engagement tied to perceived benefits, while decliners’ willingness remained tentative, hinging on the removal of logistical barriers.

## Discussion

This study demonstrates how methods in information communication including pacing, clarity, and contextualization appeared to shape how women made sense of trial information, with clearer, more accessible explanations linked to better comprehension and participation, whereas decliners frequently reported explanations that were rushed, confusing, or inadequately contextualized. This contrast mirrors broader findings from qualitative research on clinical trial recruitment, which emphasize that how trial information is delivered strongly shapes comprehension and participation decisions [44]. A recent multicenter qualitative investigation found that patients’ willingness to participate in randomized controlled trials was shaped by the way healthcare professionals communicated information, and that this communication influenced perceptions of treatment needs and potential benefits [45]. Reiterating on the importance of clear communication, evidence on informed consent shows that many trial participants lack accurate understanding of key elements such as risks, benefits, randomization, and placebo even after formal consent procedures, highlighting persistent gaps in comprehension across settings [46]. This highlights the crucial need for clear communication in clinical trials.

Building on the importance of clear information, and awareness as highlighted by our study, classic communication research in cancer trials similarly demonstrates that when trial implementation is embedded in supportive dialogue that patients and families understand, accrual decisions and patients’ feelings about those decisions are positively shaped by the quality and content of the interaction [47]. Moreover, interventions designed to improve comprehension, such as extended discussions, multimedia tools, and feedback techniques, have been shown in systematic reviews to improve understanding of procedural risks and general knowledge about studies, underscoring the malleability of comprehension when communication is deliberately structured to support it [48].

In this study, fear emerged as a common initial response to cancer screening, but its influence on participation depended on whether it was addressed through counselling and reassurance. This pattern is well documented in the literature, where fear of diagnosis, fear of bad news, and anxiety about screening procedures are consistently identified as major deterrents to screening uptake when left unresolved [49]. For instance, a qualitative study among women in cancer screening programmes showed that fear often leads to avoidance in the absence of supportive communication, particularly when individuals associate screening with inevitable bad outcomes [50]. Additionally, Vrinten et al [51], further demonstrate that fear operates bidirectionally, acting as a motivator when individuals perceive screening as a means of reassurance, but as a barrier when fear becomes overwhelming or unmediated. Broader screening literature also confirms that fear of results is one of the most persistent psychological barriers to participation and underscores the role of structured education in mitigating avoidance behaviours [52].

In this current study, acceptors framed screening as an expression of self-care and recognition of elevated health risk, particularly in the context of HIV, whereas decliners often minimized their susceptibility and saw screening as irrelevant or burdensome. Similar dynamics have been documented in the broader literature on cervical cancer screening. A Systematic review based on the Health Belief Model consistently show that high perceived susceptibility and high perceived benefits are significantly associated with uptake of screening, indicating that both recognizing personal risk and valuing the positive outcomes of screening uptake [53]. Additionally, a pooled analysis across multiple studies also found that perceived susceptibility and perceived benefits were independently associated with higher likelihood of screening uptake and intention [54]. Cross sectional research among women in clinical settings also showed that those who perceive clear benefits, such as early detection and health reassurance, alongside believing they are at risk, are more likely to participate in screening than those who do not [55].

Studies specifically among WLHIV show that greater awareness of increased vulnerability to cervical cancer correlates with higher likelihood of screening, while low perceived risk and misconceptions about susceptibility contribute to low screening adherence [56]. Additionally, research grounded in health beliefs among general female populations confirm that women who believe cervical cancer is serious and feel personally susceptible are more likely to engage in screening, whereas those with low perceived risk remain unengaged [57]. Just like in our study, where decliners often minimize their risk, a qualitative work on non-participants in screening programmes also shows that low risk perception, fatalistic beliefs, and avoidance of cancer information are linked to decisions not to attend screening, reinforcing the idea that meaning-making about risk shapes behaviour beyond awareness alone [58]. Interventional research further supports this, showing that educational programmes that increase perceived benefits also increase screening uptake, indicating that enhancing understanding of benefits and risk can be an effective strategy to drive participation [59].

Logistical barriers such as transport costs, long waiting times, and occasional shortages of screening supplies are widely recognised influences on cervical cancer screening uptake, which is similar to related barrier in implementation trials [60–62], yet their impact varies depending on how women perceive and manage these constraints.

Systematic evidence in low resource settings highlights those indirect costs, including transportation and time lost during travel and waiting, are among the most commonly reported barriers to screening participation, particularly when services are distant and time intensive to access [63, 64]. More studies across sub–Saharan Africa further showed that transport challenges and distant screening locations reduce uptake, particularly for women in rural or low-income contexts who lack affordable or reliable means of reaching services [65].

In this study, acceptors initially expressed some concern about exposure and data handling, but these worries were largely alleviated through reassurances from healthcare staff, with trust in providers creating a sense of safety and allowing participation. Decliners, by contrast, reported persistent anxiety about who might access their information and expressed broader mistrust of health staff, with privacy concerns contributing directly to refusal. This pattern aligns with evidence from other settings, where perceived breaches of confidentiality, fear of genital exposure, lack of privacy, needing spousal approval and gender of the healthcare worker were significant barriers to cervical cancer screening [66, 67]. However, broader reviews on reproductive health services confirm that mistrust in confidentiality and concerns about bodily exposure reduce engagement, highlighting the importance of creating private, respectful environments and establishing trust to support uptake [68].

In our current study, the interpersonal style and timing of recruitment interactions strongly influenced women’s willingness to participate, with acceptors describing calm, respectful, and supportive engagement as motivational, while decliners reported hurried or ill-timed approaches during moments of stress that felt intrusive or discouraging. This aligns with evidence from other screening and clinical research contexts showing that recruitment approach and social interaction significantly shape participation. Recruitment etiquette literature emphasises that politeness, respectful tone, and sensitivity to the recruiting environment contribute materially to ethical and effective participant engagement [69, 70].

However, in this study, both acceptors and decliners offered concrete ideas for improving screening delivery, with acceptors emphasising community outreach, involvement of spouses, better communication, transport support, and modest incentives, and decliners prioritising barrier reduction through early notification, language appropriate explanations, confidentiality assurances, and reduced waiting times. Both groups expressed varying degrees of openness to future participation, with acceptors’ willingness linked to perceived benefits and decliners’ conditional on resolving practical constraints. Similar evidence from low resource settings shows that women’s recommendations often Centre on addressing structural and informational barriers such as reduced costs, local language communication, female providers, and community sensitization to improve screening uptake [71].

### Study Limitations

This study has limitations that should be considered when interpreting the findings. First, data were collected through telephone interviews. While this approach was flexible and potentially reduce travel and social desirability bias, it may have excluded patients without network access and limited the richness of non-verbal cues and in-person interactions, potentially affecting the depth of qualitative insights. Second, recall bias may have influenced participants’ responses, particularly among decliners who were reflecting on decisions made during earlier ACCESS Study enrollment encounters. Third, although interviews were conducted in English, Hausa, and Yoruba and subsequently translated for analysis, subtle nuances in meaning may have been lost during translation. Finally, the study focused exclusively on WLHIV, so the findings may not be directly transferable to other populations, including HIV-negative women or men, or to trials addressing different health conditions.

Despite these limitations, participants were drawn from 12 NISA-MIRC facilities distributed across Nigeria’s six geopolitical zones, capturing a diverse range of experiences and settings, which provides valuable, context-specific insights into the decision-making processes of both acceptors and decliners in an implementation trial setting, highlighting practical and factors that influence participation.

## Conclusion

This study provides important insights into patient recruitment, refusal, and retention in implementation trials within a resource-limited setting, drawing on experiences from the ACCESS Study among WLHIV in Nigeria. Participation decisions were shaped not only by awareness or willingness, but also by emotional, cognitive, and structural factors. Women who accepted participation were influenced by clear communication, emotional reassurance, trust in healthcare providers, and perceived personal benefit, while those who declined were affected by fear, mistrust, logistical challenges, confidentiality concerns, and poorly timed recruitment encounters.

The findings highlight the importance of patient-centered recruitment approaches. However, clear, culturally appropriate communication delivered with empathy and adequate time helped build trust and reduce fear, whereas inadequate communication contributed to refusal. Structural barriers such as transportation costs, long waiting times, and competing responsibilities also influenced participation decisions.

Overall, improving recruitment and retention in clinical trials requires multifaceted strategies that address communication, trust, logistical support, and socio-cultural realities. Strengthening these areas may enhance equitable participation, improve trial implementation, and increase the generalizability of clinical research findings in Nigeria and similar settings.

## Figures and Tables

**Table 1:** Demographic Characteristics of Participants (N = 21)

Variable	Category	Acceptors (n = 12), n (%)	Decliners (n = 9), n (%)	Total (N = 21), n (%)
**Age Group (Years)**	30–39	5 (41.7%)	2 (22.2%)	7 (33.3%)
	40–49	4 (33.3%)	4 (44.4%)	8 (38.1%)
	50–59	3 (25.0%)	3 (33.3%)	6 (28.6%)
**Marital Status**	Married (Monogamous)	7 (58.3%)	6 (66.7%)	13 (61.9%)
	Married (Polygamous)	1 (8.3%)	1 (11.1%)	2 (9.5%)
	Never Married/Single	2 (16.7%)	1 (11.1%)	3 (14.3%)
	Widowed	1 (8.3%)	1 (11.1%)	2 (9.5%)
	Separated/Divorced	1 (8.3%)	0 (0.0%)	1 (4.8%)
**Education Level**	None	0 (0.0%)	1 (11.1%)	1 (4.8%)
	Primary	0 (0.0%)	1 (11.1%)	1 (4.8%)
	Secondary	1 (8.3%)	3 (33.3%)	4 (19.0%)
	Junior Secondary	0 (0.0%)	1 (11.1%)	1 (4.8%)
	Post-secondary	11 (91.7%)	3 (33.3%)	14 (66.7%)
**Religion**	Christianity	11 (91.7%)	7 (77.8%)	18 (85.7%)
	Islam	1 (8.3%)	2 (22.2%)	3 (14.3%)

**Table 2:** Comparative Thematic Summary Table: Acceptors vs Decliners

Themes	Acceptors – Sub-theme & Summary	Decliners – Subtheme & Summary	Comparative Insight
**Information, Awareness & Comprehension**	Understood information clearly; explanations were calm, paced, and empowering, enabling comprehension and confidence.	Information felt rushed, unclear, or mixed-language; led to confusion and disengagement.	Communication quality shaped comprehension: relational clarity for acceptors vs. information disconnect for decliners.
**Fear, Risk Perception & Emotional Responses**	Initial fear was managed through counselling, reassurance, and supportive framing.	Fear remained unresolved, leading to anxiety, avoidance, and withdrawal from screening.	Emotional support transformed fear for acceptors; lack of support allowed fear to dominate decliners.
**Health Motivation, Personal Relevance & Prevention Beliefs**	Screening viewed as part of self-care and essential due to perceived vulnerability.	Screening seen as irrelevant based on age, sexual inactivity, or low perceived risk.	Acceptors integrated screening into identity; decliners distanced themselves from perceived eligibility.
**Perceived Benefits & Opportunities**	Reported clear personal, social, and educational benefits; participation seen as valuable.	Benefits appeared minimal or outweighed by stress, doubt, and competing priorities.	High benefit perception promoted participation; low benefit perception encouraged refusal.
**Practical, Logistical & Time-Related Barriers**	Barriers were manageable and secondary to the importance of screening.	Barriers were decisive and made participation feel unrealistic.	Same constraints were tolerable for acceptors but prohibitive for decliners.
**Privacy, Confidentiality & Bodily Exposure Concerns**	Concerns reduced by trust and confidentiality assurances.	Mistrust about information handling and exposure.	Trust neutralized concerns for acceptors; mistrust amplified concerns for decliners.
**Recruitment Approach & Social Interaction**	Respectful, calm interpersonal approach and peer participation encouraged acceptance.	Poor timing, fatigue, and hurried interactions decreased willingness.	Tone and timing jointly influenced participation; timing failures outweighed positive tone.
**Suggestions for Improvement**	Emphasised scaling up, community outreach, inclusion of husbands, and incentives.	Focused on fixing communication, waiting time, transport support, and confidentiality.	Acceptors sought program enhancement; decliners sought barrier removal.
**Future Participation Willingness**	Strong willingness to participate again, especially with incentives and support.	Conditional willingness dependent on improved timing, transport, and privacy.	Acceptors showed enthusiastic reengagement; decliners showed tentative, conditional openness.

## Data Availability

Available and can be provided if needed. Contact: admin@ivaninstitute.org
